# A predictive nomogram for postoperative ovarian endometrioma recurrence in patients with congenital obstructive Müllerian anomalies: a retrospective study

**DOI:** 10.3389/fmed.2026.1714370

**Published:** 2026-02-26

**Authors:** Xiaotong Liu, Ning Zhang, Xuyin Zhang, Keqin Hua, Jingxin Ding, Meng Xie

**Affiliations:** 1 Department of Gynecology, Obstetrics and Gynecology Hospital of Fudan University, Shanghai, China; 2 Shanghai Key Laboratory of Female Reproductive Endocrine-Related Diseases, Shanghai, China; 3 Department of Ultrasound, Obstetrics and Gynecology Hospital of Fudan University, Shanghai, China

**Keywords:** endometrioma recurrence, long-term follow-up, obstructive Müllerian anomalies, ovarian endometrioma, predictive model

## Abstract

**Objective:**

This study aimed to develop a predictive model for ovarian endometrioma (OE) recurrence in patients with congenital obstructive Müllerian anomalies (OMAs) undergoing surgical intervention.

**Methods:**

This retrospective cohort study included 139 OMA patients with histologically confirmed ovarian endometrioma undergoing complete lesion excision and anatomical reconstruction between January 2013 and December 2020. A multivariable Cox regression analysis identified recurrence predictors; a nomogram was constructed and validated via time-dependent receiver operating characteristic curve (ROC), calibration curve, and decision curve analysis.

**Results:**

The mean surgical age of 139 patients was 20.70 ± 5.81 years. Over a mean follow-up of 80.8 months, 29.5% of patients experienced OE recurrence. Cumulative recurrence rates were 1.4% (24 months), 10.1% (36 months), 27.1% (60 months), and 34.4% (120 months). In a multivariate analysis, independent risk factors for endometrioma recurrence, such as preoperative hematometra >5 cm^3^ (hazard ratio [HR]: 2.650, 95%CI: 1.356–5.17, *p* = 0.004), rASRM score >40 (HR: 3.488, 95%CI: 1.252–9.709, *p* = 0.017), non-postoperative pregnancy (HR: 5.329, 95%CI: 1.399–20.307, *p* = 0.014), and hormonal treatment ≤30 months (HR: 3.563, 95%CI: 1.707–7.439, *p* = 0.001), and the other essential recurrent factor, surgical age, were all included in the nomogram. The nomogram showed strong discrimination (5-year AUC = 0.862, 10-year AUC = 0.808) and calibration, with decision curve analysis confirming clinical utility across probability thresholds. Internal validation via repeated K-fold cross-validation further showed robust model performance (5-year AUC = 0.864, 10-year AUC = 0.800).

**Conclusion:**

This model effectively stratifies OE recurrence risk in OMA patients post-surgery, guiding personalized management. Early surgical intervention may optimize endometrioma recurrence prevention to relieve Müllerian duct obstruction combined with prolonged postoperative medical suppression.

## Introduction

Congenital obstructive Müllerian anomalies (OMAs) result from incomplete development of the Müllerian ducts, manifesting in diverse clinical presentations (1). Müllerian anomalies encompass congenital developmental malformation and dysfunction of the uterus, the cervix uteri, and the vagina (2). Each type of malformation can be divided into obstructive and non-obstructive (3–6). Notably, complex obstructive anomalies involving multiple segments may be observed concomitantly in individual patients.

Endometriosis, characterized by the ectopic growth of endometrial-like tissue beyond the uterine cavity, predominantly affects the ovaries and the pelvic structures (7). The strong association between OMA and ovarian endometrioma (OE) is multifactorial, extending beyond the classic theory of retrograde menstruation. In patients with obstructive OMAs, the chronic outflow obstruction leads to the accumulation of menstrual blood, creating a sustained, high-pressure retrograde flow into the pelvic cavity. This not only increases the volume and frequency of endometrial cell dissemination but also establishes a unique pelvic microenvironment (8). The persistent presence of blood breakdown products induces chronic inflammation and may alter local immune surveillance, thereby facilitating the implantation, survival, and growth of ectopic endometrial tissue on the ovarian surface (9, 10). Furthermore, given that OMA often manifests at menarche, these patients experience a prolonged duration of this pathogenic process, culminating in a high prevalence of severe, recurrent OE (11). Previous studies have shown that endometriosis was significantly more common in patients with obstructive Müllerian anomalies (12). A recent systematic review and meta-analysis has reported that endometriosis prevalence was significantly higher in OMA cases (47, 95% CI: 36–58%) versus non-OMA cases (19, 95% CI: 15–24%), with an OR of 4.72 (95% CI: 2.54–8.77) (8).

Definitive surgical correction of the anatomical obstruction and the excision of endometriotic lesions is the cornerstone of management for OMA patients with OE. While surgery successfully relieves symptoms and restores anatomical continuity, it does not guarantee a cure for endometriosis. Postoperative recurrence of OE remains a significant clinical challenge (13, 14). This persistence of disease risk can be attributed to several interconnected mechanisms: (1) Incomplete surgical clearance: Achieving complete microscopic eradication of all endometriotic implants, particularly from the ovarian cortex, is technically challenging. (2) The correction of anatomical obstruction may not instantly reverse the years of chronic inflammation and altered immune surveillance established by long-standing hematometra. (3) OMA patients often present with extensive, deep-infiltrating endometriosis at a young age due to prolonged, untreated retrograde menstruation, which carries a higher risk of recurrence. Consequently, identifying which patients, despite optimal surgery, harbor the highest risk for recurrence is essential for tailoring surveillance strategies and considering prolonged adjuvant medical therapy. However, current prediction models for endometriosis recurrence are derived from general populations and fail to incorporate OMA-specific factors, for instance, preoperative hematometra and the type of anatomical reconstruction. This gap underscores the necessity to develop a dedicated predictive tool for this unique high-risk cohort. OE recurrence, especially after seemingly curative surgery, is often a late event. Shorter follow-up studies significantly underestimate the true recurrence rate and fail to capture the long-term disease course. This long-term perspective is crucial for planning the care of young OMA patients, for whom fertility preservation and decades of future health are paramount.

In summary, patients with OMA constitute a unique high-risk cohort for severe and recurrent OE, even after anatomically corrective surgery. Currently, clinicians have no OMA-specific tool to quantify this residual risk for individual patients, hindering personalized postoperative management. To translate this complex clinical scenario into actionable prognostic information, our study aimed to identify high-risk factors of OE recurrence in surgically corrected OMA and develop a predictive model using ≥5-year follow-up data to guide personalized surveillance intervals and targeted interventions.

## Materials and methods

### Participants

This retrospective cohort study enrolled patients with pathologically confirmed ovarian endometrioma and obstructive Müllerian anomalies who underwent anatomical reconstruction with complete excision of endometriotic lesions at the Obstetrics and Gynecology Hospital of Fudan University between January 2013 and December 2020. Patients were identified through a systematic search of our institutional electronic medical record system, cross-referenced with surgical procedure logs and pathology department databases.

All consecutive patients meeting the following eligibility criteria within the study period were initially screened for inclusion. The inclusion criteria were as follows: (1) The diagnosis of OMA with functioning endometrium was confirmed by magnetic resonance imaging (MRI) and intraoperative exploration; (2) histopathologically diagnosed ovarian endometrioma; (3) premenopausal status: premenopausal status was classified as women aged ≤45 years with regular menstrual cycles (21–35 days) or those with irregular cycles or on hormonal treatment, anti-Müllerian hormone (AMH) level ≥1.1 ng/mL; (4) absence of residual lesions on initial postoperative ultrasonographic assessment (≤6 weeks); and (5) complete clinical follow-up ≥40 months.

The excluded criteria comprised: (1) presence of residual endometriosis lesions postoperatively; (2) concurrent disorders of sex development (DSD); (3) postoperative follow-up duration <40 months; and (4) patients underwent hysterectomy with bilateral salpingectomy. The surgical indication for hysterectomy with bilateral salpingectomy was determined by patients with cervical and vaginal atresia who refused to accept the risk of preserving the uterus, rather than by cases involving more severe disease or surgical failure.

### Surgical procedure

All surgical procedures were performed by a specialized team of senior gynecological surgeons, each with over 15 years of experience in complex benign gynecologic surgery and Müllerian anomaly reconstruction prior to the study period. While surgical instruments and energy devices evolved over 8 years, the fundamental principles and standardized steps of the procedure remained unchanged. All surgeons adhered to a unified, institutionally approved surgical protocol: laparoscopic metroplasty for hemi-uterus with a functional non-communicating rudimentary cavity (3); hysteroscopic obstruction relief for Robert’s uterus (4); cervicovaginal reconstruction with cervical stent placement for cervical and vaginal atresia (5); and transvaginal septum resection for transverse vaginal septum/HWWS (6). For patients undergoing hysteroscopic or cervicovaginal reconstruction, the anatomical patency of the outflow tract was routinely verified intraoperatively via hysteroscopy or by a cervical dilator.

Concurrently, the systematic laparoscopic eradication of endometriosis was achieved; the overarching surgical principle was the complete excision of the endometriotic cyst wall with the maximum preservation of the normal ovarian cortex: (1) precision cortical incision along the ovarian hilum: the cleavage plane between the fibrotic cyst wall and the ovarian stroma was precisely identified using atraumatic grasping forceps; (2) forceps were used to strip the endometrioma from the ovarian parenchyma; if integral removal was not feasible, the cyst was opened, the interior was inspected, and the lining was meticulously stripped in fragments, with care to avoid leaving visible pigmented tissue. Coagulation is performed only on pinpoint bleeding vessels to avoid broad thermal damage to the ovarian hilus; (3) complete excision/ablation of all visible peritoneal and deep infiltrating lesions. All patients received postoperative oral contraceptives to suppress ovulation and retrograde menstruation. In our protocol, agents such as GnRH agonists and dienogest were specifically excluded. This deliberate choice was made because oral contraceptives, in contrast to the prolonged amenorrhea induced by GnRH agonists or dienogest, allow for a clearer assessment of menstrual resumption. The timely return of menses served as a critical clinical indicator of surgical success and the absence of recurrent outflow tract obstruction.

### Data collection

Obstructive Müllerian anomalies were classified into uterus, cervix, and vagina anomalies according to the European Society of Human Reproduction and Embryology (ESHRE)/European Society for Gynaecological Endoscopy (ESGE) criteria (2). Clinical data were systematically extracted by an independent researcher: (1) Demographics: surgical age. (2) Symptoms: preoperative cyclic abdominal pain duration. Preoperative cyclic pain and postoperative dysmenorrhea severity were quantified using VAS scores. To standardize reporting, patients were instructed to rate their worst pain experienced during menstruation. (3) Biomarkers: preoperative serum cancer antigen 125 (CA125) and anti-Müllerian hormone (AMH) levels. Serum CA125 levels were measured from blood samples drawn during the early follicular phase, whenever possible, to minimize cyclical variation. For patients with amenorrhea due to obstruction, the sample was drawn during cyclic abdominal pain. AMH levels were measured using the electrochemiluminescence immunoassay and expressed as ng/mL. (4) Imaging: endometrioma size and preoperative hematometra volume. The size of the endometrioma was determined by measuring the maximum cyst diameter (cumulative diameters for bilateral cases). Preoperative hematometra volume serves as a direct, quantifiable surrogate for the severity of the obstructive phenomenon, which is quantitatively assessed using pelvic magnetic resonance imaging. The volume was calculated using the ellipsoid formula based on measurements of the fluid-filled uterine cavity in three orthogonal planes. (5) Surgical documentation: adenomyosis/leiomyoma, diagnosed with characteristic preoperative imaging findings or histopathological confirmation from surgical specimens; deep infiltrating endometriosis (DIE); the revised American Society for Reproductive Medicine (rASRM) score (15); and the type of obstructive genital tract anomalies. (6) Outcomes: postoperative pregnancy rates, hormonal therapy durations, and recurrence intervals. Postoperative pregnancy was defined as the achievement of clinical pregnancy regardless of conception method (spontaneous or through assisted reproductive technology).

### Follow-up and definition of recurrence

Our institution implements a standardized surveillance protocol comprising pelvic exams and transvaginal ultrasound at 3-month intervals during the first postoperative semester (0–6 months), extended to 6-month or annual intervals for asymptomatic patients. Compliance with the follow-up schedule was promoted through a combination of scheduled outpatient clinic visits, dedicated nurse-led telephone reminders, and coordination with referring physicians. OE recurrence was defined as the sonographic or surgical identification of a new endometrioma on either the ipsilateral ovary or the contralateral ovary. Standardized sonographic criteria: (1) ground-glass echogenicity; (2) 2–4 locules with diameter ≥2 cm; (3) low vascularization without papillae; (4) persisting through ≥3 menstrual cycles to differentiate from transient functional cysts (16, 17). For the purpose of applying the persistence over ≥3 menstrual cycles criterion, a standard menstrual cycle was defined as 21–35 days in patients with regular cycles. For patients with irregular cycles (>35 days or <21 days), the actual interval between their last three menstrual periods was used to calculate a personalized cycle length. For patients who were amenorrheic, a cyst was required to be persistent or enlarging over a minimum interval of 84 days (equivalent to three 28-day cycles) on serial ultrasound examinations. To minimize observer bias, sonographers were blinded to the patient’s clinical history, surgical details, and previous ultrasound reports. All ultrasound examinations were performed according to a standardized institutional protocol by experienced sonographers. While not all scans were performed by a single examiner, all sonographers underwent regular quality assurance calibration, and ambiguous findings were reviewed by a designated senior radiologist specializing in gynecologic imaging. The recurrence interval was calculated from the date of definitive surgery to imaging-confirmed recurrence.

### Ethical approval and informed consent

The methodology for this study was approved by the Institutional Review Board of the Obstetrics and Gynecology Hospital of Fudan University (Ethics approval number: 2022-47). All included patients or their legal guardians provided consent to research use of their medical data.

### Statistical analysis

Statistical analyses used SPSS 26.0 and R 4.0.0. Quantitative variables were compared using appropriate parametric/non-parametric tests, and categorical variables were compared using the χ^2^/Fisher’s exact tests. Kaplan–Meier survival analysis with log-rank testing assessed recurrence probabilities. Univariate and multivariate analyses of the Cox proportional hazards regression model were incorporated to determine recurrence-related factors. Predictor variables for the Cox proportional hazards regression model were pre-specified based on possible related factors of clinical and pathophysiological relevance to OE recurrence in OMA patients. A univariate Cox regression analysis was first performed on these candidate variables; variables with a significance level of *p*-value < 0.10 in the univariate analysis were retained for consideration in the subsequent multivariate model. This threshold was chosen to avoid prematurely excluding potentially important predictors. The final multivariable Cox model identified independent predictors of recurrence (*p* < 0.05). The nomogram constructed via the R’s “rms” package included all significant predictors from the multivariable Cox model (*p* < 0.05). The nomogram construction involved the following steps: First, prognostic predictors were weighted according to their respective HRs, with higher HR values translating into proportionally scaled point allocations. Subsequently, these weighted components were systematically aligned using a standardized coordinate system, creating a multi-axis graphical interface. Time-dependent area under the curve (AUC) (“survivalROC”), bootstrap-calibrated curves, and decision curve analysis evaluated model performance. To comprehensively assess model robustness in our limited sample, we additionally performed internal validation using 5-repeated 5-fold cross-validation. The choice of K = 5 ensured each validation fold retained an adequate sample size for reliable estimation while maximizing training data utilization—a critical consideration for rare disease cohorts. Statistical significance was set at a *p*-value of < 0.05.

## Results

**Figure 1:**
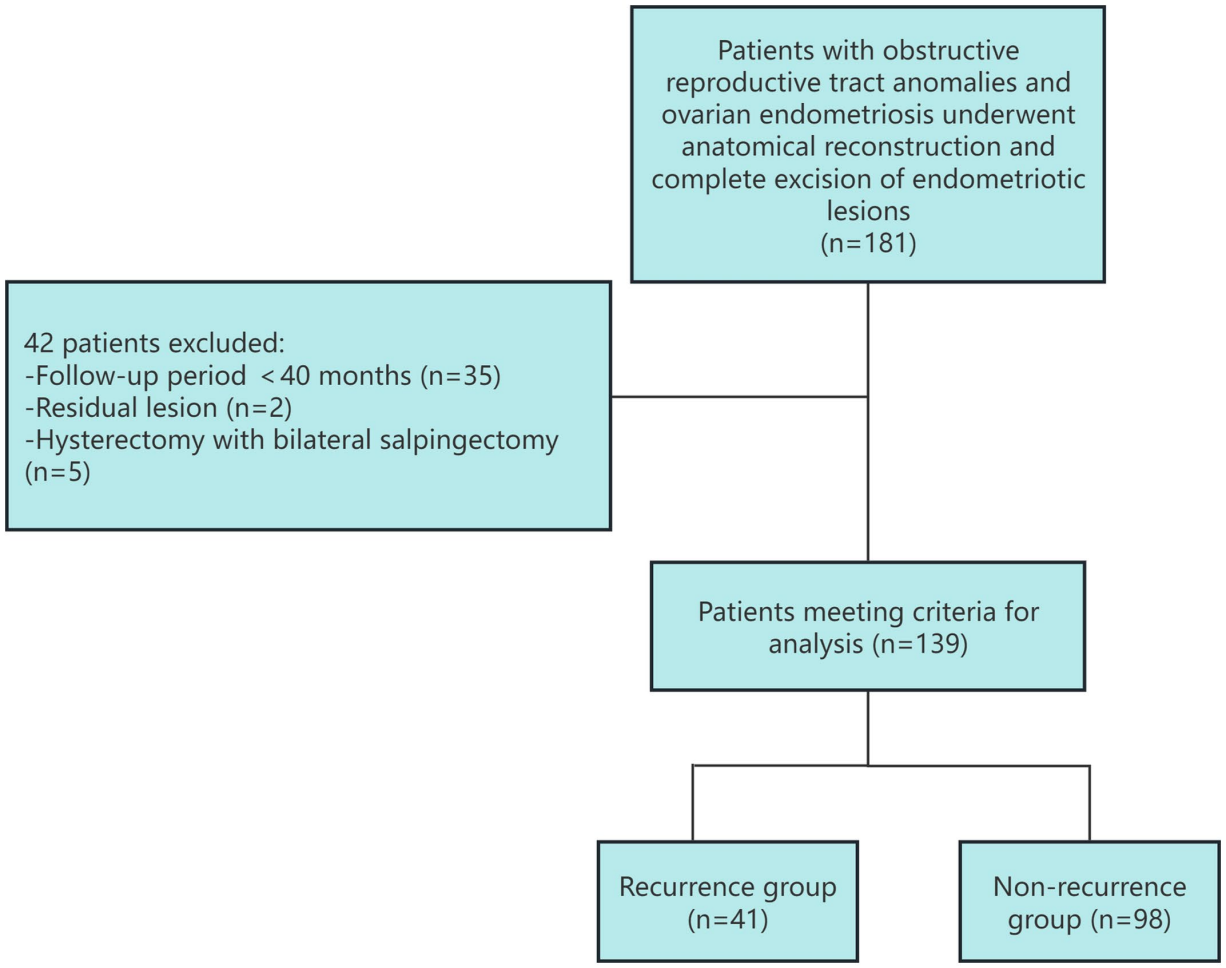
Flow diagram of the patient selection process.

Of the 181 patients initially identified, 42 were excluded: 35 for a follow-up period of <40 months, 2 for a residual endometriosis lesion, and 5 for hysterectomy with bilateral salpingectomy. Thus, 139 patients constituted the final study cohort (Figure 1). The mean surgical age of 139 patients was 20.70 ± 5.81 years. The mean follow-up time was 80.79 ± 27.15 (40–131) months. While all cases achieved initial pain relief, seven cases required reoperation for outflow tract re-obstruction (mean: 19.2 months postoperatively). Meanwhile, 37 (26.62%) patients were confirmed to have successful delivery.

### Recurrence characteristics of the patients

**Figure 2:**
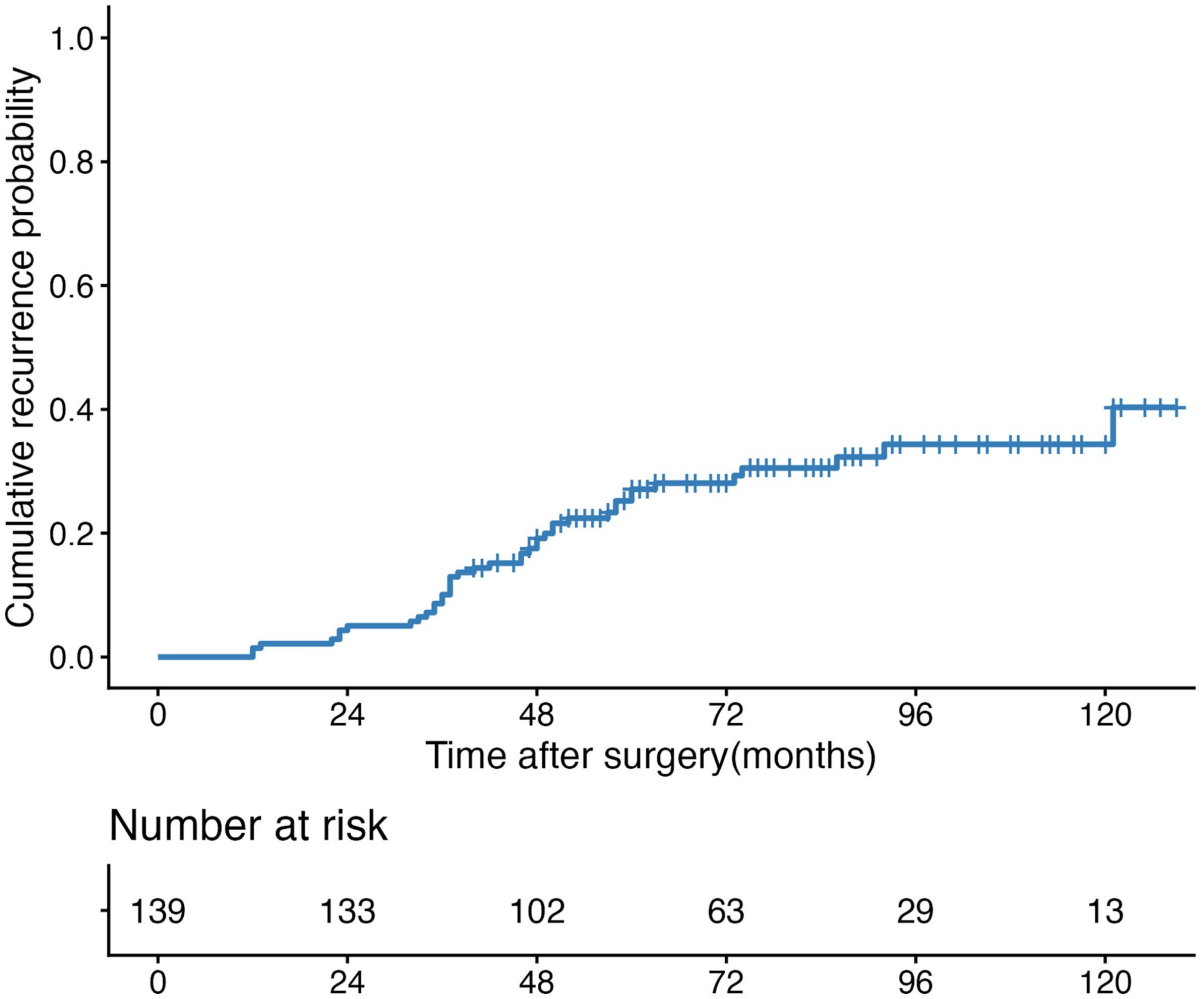
Kaplan–Meier curve of cumulative recurrence rates of ovarian endometrioma.

Ovarian endometrioma recurred in 41 patients (29.5%). The cumulative recurrence rates of ovarian endometrioma at 24 months, 36 months, 60 months, and 120 months were 1.4% (95% CI: 0.7–3.4%), 10.1% (95% CI: 4.9–14.9%), 27.1% (95% CI, 18.9–34.5%), and 34.4% (95% CI, 24.2–43.1%), respectively (Figure 2). The median recurrence-free survival was not reached during the follow-up period.

The clinical characteristics of patients (*n* = 139) with and without ovarian endometrioma recurrence are summarized in Table 1. Recurrence cases showed significantly prolonged cyclic pain duration, elevated VAS scores, higher preoperative CA125 levels, larger hematometra, increased endometrioma size, bilateral cyst predominance, a more advanced rASRM stage IV, a reduced postoperative pregnancy rate, and a shorter duration of postoperative hormonal therapy (all *p* < 0.05).

**Table 1:** Clinical characteristics of the ovarian endometrioma recurrence and non-recurrence groups.

Variables	All (*n* = 139)	Endometriosis recurrence		*p*-value
		Yes (*n* = 41)	No (*n* = 98)	
Surgical age (years)	20.70 ± 5.81 (11–33)	19.68 ± 6.01 (12–33)	21.13 ± 5.69 (11–32)	0.18
Cyclic abdominal pain (months)	27.93 ± 34.40 (0–240)	42.29 ± 49.39 (6–240)	21.92 ± 23.54 (0–120)	0.001*
VAS scores before surgery	7.24 ± 2.04 (0–10)	8.10 ± 1.28 (4–10)	6.89 ± 2.19 (0–10)	<0.001*
Preoperative CA125 (IU/mL)	56.62 ± 24.39 (5.73–126.53)	67.01 ± 26.71 (23.64–126.53)	52.27 ± 22.08 (5.73–110.65)	0.002*
Preoperative AMH (ng/mL)	5.20 ± 1.21 (2.05–8.90)	5.09 ± 1.21 (2.91–7.90)	5.25 ± 1.21 (2.05–8.90)	0.475
Hematometra before surgery (cm^3^)	4.02 ± 3.03 (0–12)	5.76 ± 3.06 (0–12)	3.30 ± 2.71 (0–9)	<0.001*
Endometrioma size (cm)	6.99 ± 3.34 (2–22)	8.90 ± 3.33 (2–15)	6.19 ± 3.01 (2–22)	<0.001*
Laterality				
Unilateral	108 (77.70%)	25 (23.15%)	83 (76.85%)	0.002*
Bilateral	31 (22.30%)	16 (51.61%)	15 (48.39%)	
Leiomyoma				
Yes	17 (12.23%)	6 (35.29%)	11 (64.71%)	0.576
No	122 (87.77%)	35 (28.69%)	87 (71.37%)	
Adenomyosis				
Yes	18 (12.95%)	3 (16.67%)	15 (83.33%)	0.201
No	121 (87.05%)	38 (31.40%)	83 (68.60%)	
Deep infiltrating endometriosis				
Yes	10 (7.19%)	4 (40%)	6 (60%)	0.481
No	129 (92.81%)	37 (28.68%)	92 (71.32%)	
rASRM score	46.66 ± 24.28 (6–106)	63.41 ± 27.25 (12–106)	39.65 ± 19.09 (6–88)	<0.001*
Stages I–III	64 (46.04%)	9 (14.06%)	55 (85.94%)	<0.001*
Stage IV	75 (53.96%)	32 (42.67%)	43 (57.33%)	
Obstructive type				
Uterine anomalies	71 (51.08%)	20 (28.17%)	51 (71.83%)	0.078
Cervical anomalies	35 (25.18%)	15 (42.86%)	20 (57.14%)	
Vaginal anomalies	33 (23.74%)	6 (18.18%)	27 (81.82%)	
Postoperative re-obstruction				
Yes	7 (5.04%)	4 (57.14%)	3 (42.86%)	0.195
No	132 (94.96%)	37 (28.03%)	95 (71.97%)	
Dysmenorrhea after surgery				
Yes	51 (36.69%)	18 (35.29%)	33 (64.71%)	0.254
No	88 (63.31%)	23 (26.14%)	65 (73.86%)	
Postoperative pregnancy				
Yes	37 (26.62%)	3 (8.11%)	34 (91.89%)	0.001*
No	102 (73.38%)	38 (37.25%)	64 (62.75%)	
Hormonal treatment duration before recurrence (months)	31.06 ± 13.26 (6–60)	25.27 ± 9.59 (6–42)	33.48 ± 13.87 (6–60)	0.003*
0–12	16 (11.51%)	9 (56.25%)	7 (43.75%)	0.004*
13–24	34 (24.46%)	10 (29.41%)	24 (70.59%)	
25–36	55 (39.57%)	19 (34.55%)	36 (65.45%)	
37–48	19 (13.67%)	3 (15.79%)	16 (84.21%)	
49–60	15 (10.79%)	0	15 (100%)	

VAS, visual analog scale; AMH, Anti-Müllerian Hormone; rASRM, revised American Society for Reproductive Medicine.

*Significant results.

### Independent prognostic factors in the derivation cohort

To objectively define a clinically applicable threshold for the baseline predictor preoperative hematometra volume, a conventional receiver operating characteristic curve (ROC) curve analysis was performed. The analysis yielded an area under the curve (AUC) of 0.72 for hematometra volume in predicting recurrence. The optimal cutoff value, as determined by maximizing the Youden index, was 5.4 cm^3^, with a corresponding sensitivity of 56% and a specificity of 79%. Given the proximity of this statistically optimal value to the clinically relevant threshold of 5 cm^3^, a cutoff of >5 cm^3^ was retained for inclusion in the subsequent analysis.

The Cox regression analysis identified independent recurrence predictors (Table 2). The univariate analysis revealed 11 significant predictors (*p* < 0.1): surgical age ≤ 20 years, preoperative cyclic abdominal pain >18 months, preoperative VAS scores > 8, preoperative CA125 > 64 IU/mL, preoperative hematometra > 5 cm^3^, endometrioma size > 7 cm, bilateral ovarian endometrioma, rASRM score > 40, postoperative obstruction, no postoperative pregnancy, and hormonal treatment ≤ 30 months. All these variables were selected for further multivariate COX regression analysis. The results indicated that preoperative hematometra >5 cm^3^ (HR: 2.650, 95%CI: 1.356–5.17, *p* = 0.004), rASRM score >40 (HR: 3.488, 95%CI: 1.252–9.709, *p* = 0.017), non-postoperative pregnancy (HR: 5.329, 95%CI: 1.399–20.307, *p* = 0.014), and hormonal treatment ≤ 30 months (HR: 3.563, 95%CI: 1.707–7.439, *p* = 0.001) were significant risk factors for ovarian endometrioma recurrence in the multivariate analysis.

**Table 2:** Univariate and multivariate COX analyses of risk factors of recurrent ovarian endometrioma in patients with obstructive genital tract anomalies.

Factors	Univariate analysis		Multivariate analysis	
	HR (95% CI)	*p*	HR (95% CI)	*p*
Surgical age (≤ 20 years vs. >20 years)	2.061 (1.088–3.907)	0.027*	1.508 (0.697–3.326)	0.297
Cyclic abdominal pain (> 18 months vs. ≤ 18 months)	2.186 (1.145–4.175)	0.018*	0.970 (0.410–2.296)	0.945
Preoperative VAS scores (> 8 vs. ≤8)	2.343 (1.263–4.344)	0.001*	1.058 (0.515–2.171)	0.878
Preoperative CA125 (> 64 IU/mL vs. ≤ 64 IU/mL)	2.020 (1.094–3.729)	0.025*	1.557 (0.774–3.132)	0.214
Preoperative AMH (> 5 ng/mL vs. ≤ 5 ng/mL)	0.824 (0.446–1.524)	0.538		
Preoperative hematometra (> 5cm^3^ vs. ≤ 5 cm^3^)	2.910 (1.568–5.400)	0.001*	2.650 (1.356–5.175)	0.004*
Endometrioma size (> 7 cm vs. ≤ 7 cm)	2.919 (1.545–5.515)	0.001*	2.376 (0.885–6.382)	0.086
Ovarian endometrioma (Bilateral vs. Unilateral)	2.645 (1.410–4.960)	0.002*	0.729 (0.302–1.757)	0.481
Leiomyoma (yes vs. no)	0.855 (0.359–2.034)	0.723		
Adenomyosis (yes vs. no)	2.162 (0.667–7.012)	0.199		
DIE (yes vs. no)	1.870 (0.665–5.259)	0.235		
rASRM score (> 40 vs. ≤ 40)	3.479 (1.6585–7.297)	0.001*	3.488 (1.252–9.709)	0.017*
Obstructive type (uterine or cervical anomalies vs. vaginal anomalies)	1.343 (0.870–2.074)	0.183		
Postoperative obstruction (yes vs. no)	3.556 (1.260–10.032)	0.017*	1.526 (0.461–5.052)	0.499
Dysmenorrhea after surgery (yes vs. no)	1.325 (0.715–2.456)	0.372		
Postoperative pregnancy (no vs. yes)	6.087(1.874–19.768)	0.003*	5.329 (1.399–20.307)	0.014*
Hormonal treatment (≤ 30 months vs. > 30 months)	1.991 (1.015–3.906)	0.045*	3.563 (1.707–7.439)	0.001*

VAS, visual analog Scale; AMH, Anti-Müllerian Hormone; DIE, Deep infiltrating endometriosis; rASRM, revised American Society for Reproductive Medicine.

*Significant results.

### Nomogram for predicting recurrence

**Figure 3:**
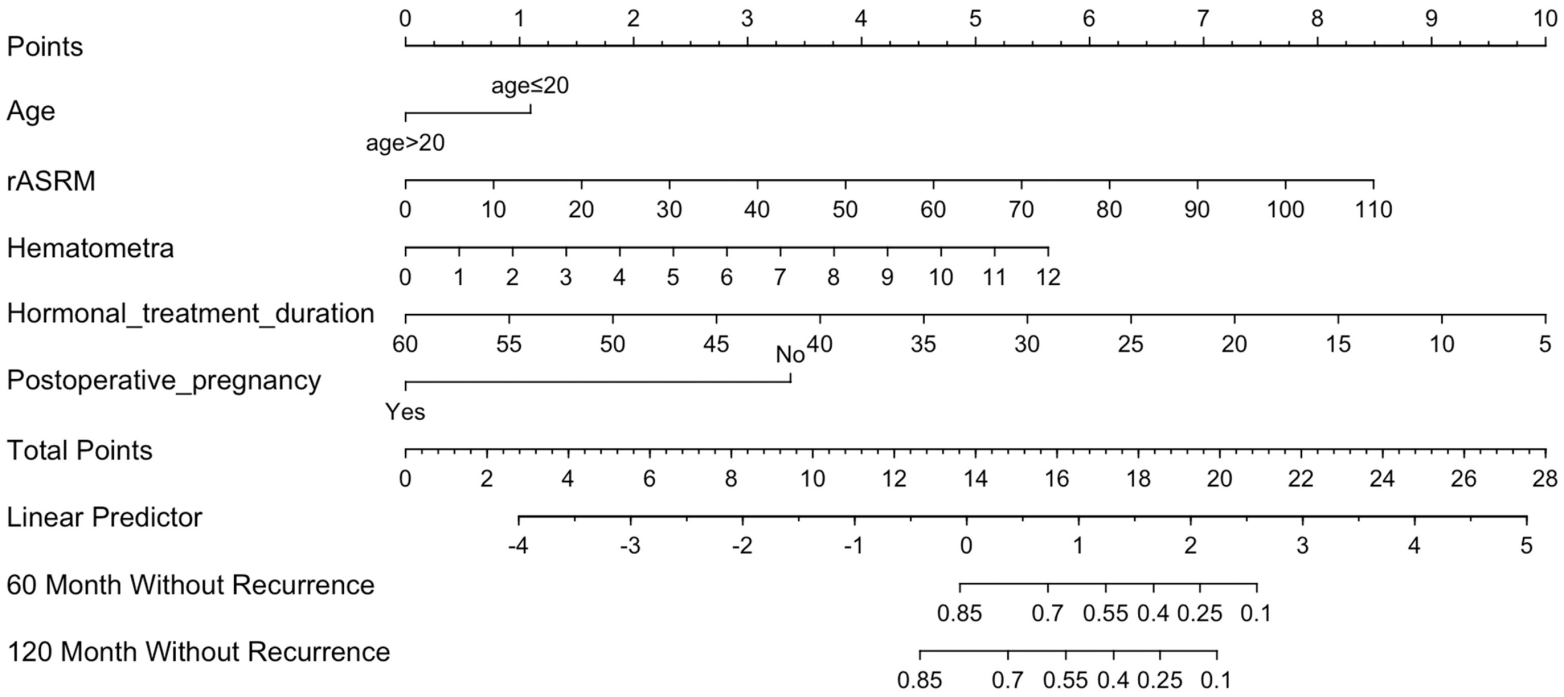
Nomogram for predicting ovarian endometrioma recurrence in patients with obstructive genital tract anomalies. To apply the nomogram, first identify patient-specific values on each variable axis and plot vertical lines to determine corresponding point allocations. Accumulate these points on the “Total Points” axis and then project a horizontal line to the survival probability axis to estimate 60- or 120-month recurrence-free survival probabilities.

The nomogram incorporated multivariable Cox predictors (*p* < 0.05, five independent predictors) and clinically significant age (Figure 3). This visual prediction tool enabled clinicians to calculate individualized 60- and 120-month recurrence-free probabilities through cumulative score summation and bottom-scale projection based on personal clinical parameters.

### Validation of the nomogram

**Figure 4:**
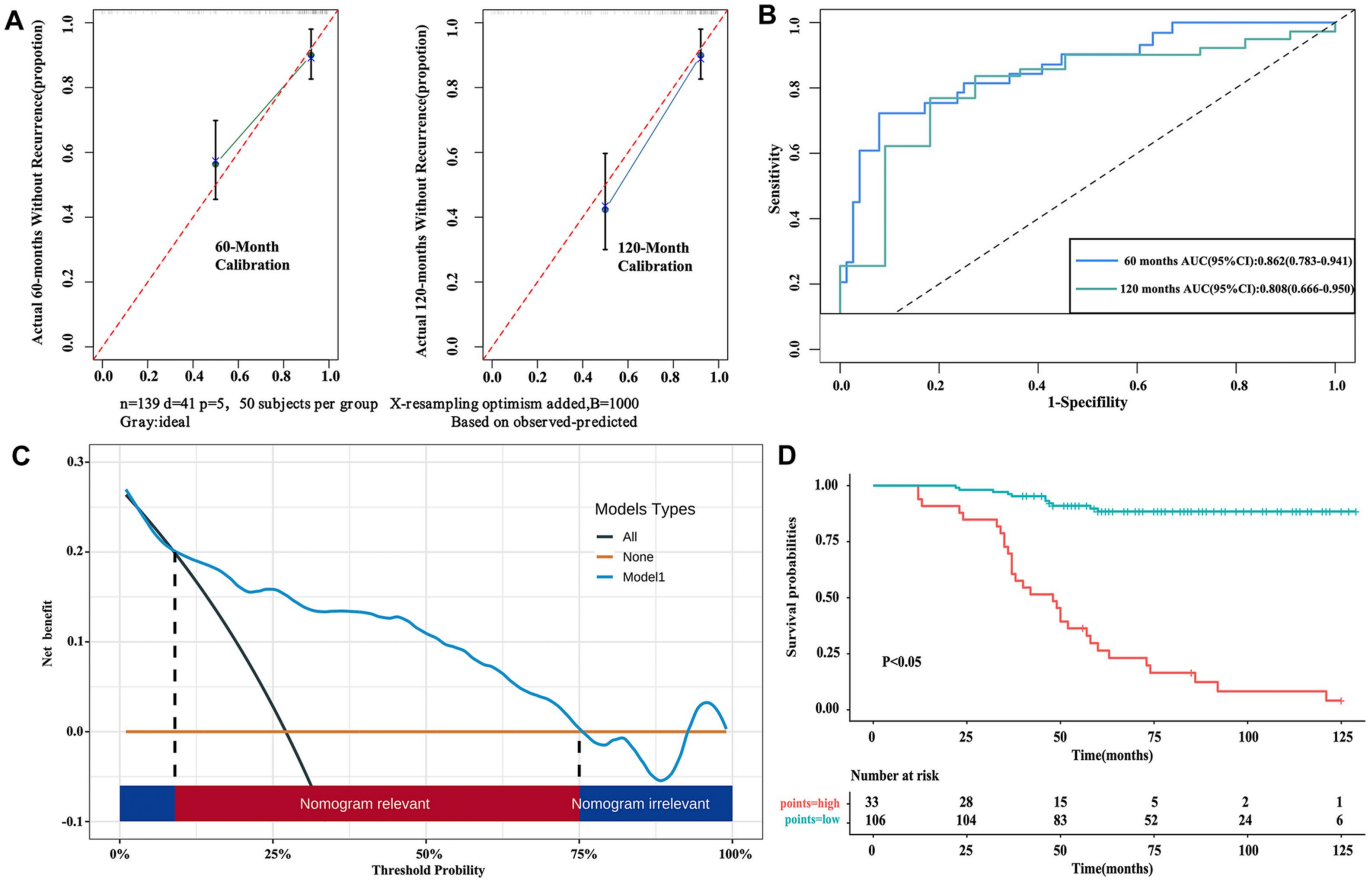
Validation of the nomogram. **(A)** The calibration curve for predicting patients without recurrence at 60 months (left) and 120 months (right) in the derivation cohort. **(B)** The area under the curve (AUC) of the nomogram predictive model at 60 months and 120 months in the derivation cohort. **(C)** The decision curve analysis (DCA) of the nomogram. **(D)** Comparison of the prognosis for the low-risk and high-risk groups defined by our predictive model.

Internal validation via bootstrap resampling (*n* = 1,000) and repeated K-fold cross-validation confirmed model consistency and robustness. The calibration curves for postoperative recurrence-free survival probabilities revealed excellent concordance between predicted and observed outcomes at both 60- and 120-month follow-ups (Figure 4A). The receiver operating characteristic curve (ROC) analysis revealed strong discriminative performance of the model, with an area under the curve (AUC) of 0.862 (95% CI: 0.783–0.941) for 60-month recurrence-free survival and 0.808 (95% CI: 0.666–0.950) for 120-month recurrence-free survival (Figure 4B). The decision curve analysis confirmed clinical utility (Figure 4C). Using the Kaplan–Meier curve comparison, the high-risk population exhibited significantly greater recurrence likelihood than their low-risk counterparts (*p* < 0.05), as visualized in Figure 4D.

To further address the need for rigorous validation in small, heterogeneous datasets, we supplemented the bootstrap validation with 5-repeated 5-fold cross-validation. This procedure generated 25 independent performance estimates. The model exhibited consistent and stable performance across all folds (Figure S1). The mean C-index was 0.802 ± 0.054 (95% CI: 0.778–0.826), with a coefficient of variation (CV) of 6.7%, indicating high discriminative stability. The mean time-dependent AUC for 60-month recurrence-free survival was 0.864 ± 0.075 (95% CI: 0.832–0.897; CV: 8.7%). For the 120-month prediction, the mean AUC was 0.800 ± 0.146 (95% CI: 0.735–0.864; CV: 18.3%), reflecting maintained but more variable long-term accuracy, as expected. Performance metrics showed minimal fluctuation with changes in validation set sample size and event count (Supplementary Figure S1), further supporting the model’s robustness against data partitioning variability. The concordant results from bootstrap and repeated K-fold cross-validation strengthened the internal validity and generalizability of the nomogram.

## Discussion

Congenital obstructive Müllerian anomalies with functional endometrium are rare disorders often complicated by secondary endometriosis due to retrograde menstruation (12). Despite surgical correction of anatomical obstructions, endometrioma outcomes remain unpredictable, with reports suggesting either spontaneous resolution or persistent disease following surgical intervention (14). Notably, the recurrence patterns and risk stratification of ovarian endometrioma in OMA patients remain poorly characterized. In this study, we conducted a retrospective cohort study analyzing recurrence rates and prognostic factors in OMA patients with endometrioma. Using the multivariate Cox regression analysis, we developed the first clinical nomogram specifically designed to predict long-term recurrence probabilities following surgical management. This tool enables individualized risk stratification, facilitating targeted surveillance and early intervention for high-risk subgroups. To the best of our knowledge, this investigation constitutes the largest cohort with extended follow-up examining endometrioma recurrence in this unique patient population.

The 5-year cumulative recurrence rate of 27.1% in our OMA cohort defines the recurrence risk profile for this unique population. The substantial recurrence risk that persists despite complete anatomical correction is a critical observation. It underscores that surgical intervention, while resolving the mechanical obstruction and removing visible disease, does not fully reset the pathogenic pelvic ecosystem established over years of high-pressure retrograde menstruation. The recurrence pattern in OMA patients stems from their unique disease origin: sustained retrograde menstruation creates a severe and chronic inflammatory pelvic environment from menarche. Even after the obstructive source is removed, this microenvironment may facilitate the survival of microscopic disease remnants or enhance the implantation potential of new refluxed endometrial cells (18). Our cohort analysis is the first to identify preoperative hematometra exceeding 5 cm^3^ as an independent predictor of OE recurrence (HR: 2.650, 95% CI: 1.356–5.175), aligning with the theory of retrograde menstruation and ectopic implantation. To objectively validate this threshold, we performed a conventional ROC curve analysis, which yielded an optimal data-driven cutoff of 5.4 cm^3^ based on the Youden index. The close agreement between this statistically derived value (5.4 cm^3^) and the clinically used rounded threshold (5 cm^3^) substantiates its validity. The association between preoperative hematometra volume and recurrence risk likely operates through these two ways. First, a larger hematometra implies a greater reservoir for retrograde menstruation, potentially leading to a higher volume of endometrial cells being refluxed into the peritoneal cavity with each cycle. Second, persistent hematometra is a marker of prolonged duration of obstruction. This extended timeline allows for the establishment of a more entrenched, chronic inflammatory environment in the pelvis that not only facilitates initial implantation but also may promote the survival and growth of residual microscopic disease post-surgery. This emphasizes the need for adjuvant strategies targeting the residual inflammatory environment in addition to anatomical correction.

Postoperative hormonal suppression is a cornerstone of adjuvant therapy to mitigate recurrence risk (19). In accordance with prevailing clinical guidelines, combined oral contraceptives (COCs) were used as the first-line regimen for all patients in our cohort (20). This protocolized choice was guided by their established efficacy, favorable safety profile for long-term use in young patients, and—critically for surgical follow-up in OMA patients—their predictable, cyclic withdrawal bleeding pattern. This pattern facilitates the clinical assessment of menstrual resumption, serving as a practical indicator of restored anatomical patency, which is a central concern after obstructive anomaly correction. Our findings contribute to the ongoing refinement of the optimal treatment duration. Choi et al. identified hormonal therapy ≤15 months as a significant recurrence risk factor (HR: 2.869, *p* < 0.001) (21). Our study extends this temporal relationship to OMA patients. We found that in this cohort, a postoperative hormonal therapy duration of less than 30 months was associated with a threefold higher hazard of recurrence (HR: 3.563, *p* < 0.001) compared to longer use. This suggests that the threshold for a protective duration may be substantially longer in OMA patients than in the general endometriosis population. The current ESHRE guideline, which suggests considering postoperative hormonal treatment for 18–24 months to prevent recurrence (22), may therefore be insufficient for these patients. The chronic, high-pressure inflammatory environment established preoperatively in OMA patients likely represents a more aggressive disease baseline, necessitating a more prolonged period of ovarian suppression to mitigate the risk of lesion reactivation. However, it is critical to emphasize that this association is observational and does not establish causality. Thus, our data advocate not for a rigid 30-month rule but for a paradigm of individualized, extended adjuvant treatment for OMA patients, with therapy duration tailored to individual risk profiles, tolerability, and reproductive plans. The strong association of hormonal treatment ≤30 months with recurrence is best understood as a self-fulfilling prophecy embedded in clinical practice. It identifies, from the outset, a patient profile in which achieving long-term therapeutic suppression is predictably difficult—often because the underlying disease is more aggressive. Therefore, this variable acts as a powerful prognostic integrator. It encapsulates preoperative disease severity, intraoperative findings, and postoperative treatment into an available data point. Its high hazard ratio thus reflects its efficiency in flagging a high-risk clinical phenotype where disease activity and management challenges converge.

The clinical applicability of our nomogram is supported by the decision curve analysis (DCA), which exhibited a positive net benefit across a threshold probability range of approximately 10 to 75% when compared to “treat-all” or “treat-none” strategies. This indicates that the model holds practical utility for risk stratification in clinical decision-making, particularly in tailoring postoperative management. For instance, patients identified as high-risk (e.g., with a nomogram-predicted recurrence probability >50%) could be advised to undergo extended postoperative hormonal suppression (>30 months) and intensified surveillance through transvaginal ultrasound every 6 months. Conversely, those classified as low-risk may be adequately managed with standard annual follow-up. By facilitating such individualized risk stratification, our model supports a move away from a uniform management approach toward one that personalizes both follow-up intervals and adjuvant therapy duration, thereby optimizing resource allocation and improving long-term outcomes.

In our multivariable model, a specific OMA anatomical subtype was not retained as an independent recurrence factor after accounting for other clinical variables. This suggests that its apparent effect on recurrence may be mediated through these other, more quantifiable factors. For instance, a uterine obstructive anomaly may present with a larger preoperative hematometra and more extensive endometriotic disease. This finding appears to contrast with the report by Kapczuk et al., which indicated that uterine anomalies were associated with a higher recurrence rate (12). Several factors may explain this apparent discrepancy. First, the definition and analysis of recurrence may differ. Our study used imaging-based criteria with a long-term follow-up, while other studies might use different endpoints. Second, the statistical approach matters. Our model included preoperative hematometra: a continuous, mechanistic proxy for obstruction severity, which may have captured the pathogenic essence of the anomaly subtype better than a categorical variable.

Existing evidence consistently identifies younger age as a critical determinant of postoperative endometriosis recurrence (23). While prior research established 35 years as a significant threshold for endometrioma recurrence risk (24), Choi et al. identified ≤31 years (HR: 2.108, *p* < 0.001) through a multivariable analysis of 756 patients undergoing laparoscopic surgery (21). Similarly, our findings revealed significantly younger surgical ages in recurrent cases, with ≤20 years predicting recurrence (HR: 2.061, *p* = 0.027). Although there was no significance in the multivariate modeling (*p* > 0.05), age was retained in our predictive model due to its established clinical relevance for endometriosis recurrence. Similarly, in a 2022 study by Zhiyue Gu et al. on modeling endometrioma recurrence before the age of 45 years, age was also retained in the final model due to its clinical relevance, despite lacking statistical significance in the multivariable analysis (25). While the inclusion of age may enhance the face validity and clinical applicability of the model, its independent predictive contribution in our specific cohort appears limited.

In the multivariable analysis by Tobiume et al. (26), the rASRM score was an independent factor associated with recurrence (HR: 1.0263, *p* < 0.05). Liu et al. specifically associated total rASRM score—though not staging—with recurrence risk (HR 1.012, *p* < 0.05) (27). Combined with our findings, the predictive value of rASRM scoring systems has been further corroborated. In severe stages of endometriosis characterized by infiltration and invasion of the ovarian cortex, the depth of tissue involvement creates surgical challenges in achieving complete lesion clearance, thereby predisposing to disease recurrence (28). The study protocol mandated complete lesion excision as a surgical objective, defined as the macroscopic removal of all visible endometriotic implants and ovarian cyst walls. However, we acknowledge that surgical completeness is inherently subjective and was not quantified using standardized peritoneal scoring systems. Therefore, while we support the principle that anatomical correction combined with meticulous macroscopic resection forms the critical foundation for management, prospective studies incorporating quantitative surgical scoring are needed to definitively establish its independent role.

Although preoperative AMH levels were not associated with recurrence in our cohort—suggesting that the drivers of recurrence may be independent of quantitative ovarian reserve—AMH remains a crucial biomarker reminding surgeons that any intervention in these young patients should prioritize the protection of residual ovarian function. In reproductive-age patients with ovarian endometrioma, preserving postoperative fertility is an important objective. Endometriosis itself is associated with significantly reduced pregnancy rates compared to unexplained infertility, underscoring the need to protect reproductive potential from the outset (29). This imperative is further emphasized by evidence that repeat ovarian surgery for recurrent disease can lead to a measurable decline in antral follicle count, progressively compromising ovarian reserve (30). Therefore, achieving maximal efficacy during the primary surgical intervention is critical not only for disease control but also as a proactive strategy for fertility preservation by minimizing the need for reoperation. For patients identified as high-risk for recurrence—potentially through predictive tools like the nomogram presented in this study—adjuvant fertility preservation strategies, such as ovarian tissue cryopreservation, may be considered as a proactive safeguard before surgery (31).

Koga et al. reported that subsequent pregnancy may have a protective effect on endometrioma recurrence (32). Our model identifies failure to achieve pregnancy postoperatively as a marker of elevated recurrence risk. We interpret this not as a direct biological effect of the gravid state itself, but rather as a marker of prolonged amenorrhea and the cessation of retrograde menstruation—the primary driver of disease in OMA patients. For patients pursuing immediate pregnancy, our data support actively attempting conception post-surgery, as the achieved state of prolonged physiological amenorrhea may create a critical recurrence-free window. This finding underscores the potential importance of postoperative medical suppression of ovulation in patients not pursuing immediate pregnancy as a strategy to mimic the protective amenorrheic window associated with pregnancy.

This study had some limitations. First and foremost, our nomogram was developed and internally validated within a single-center cohort. External validation is imperative to confirm its generalizability and clinical utility before it can be recommended for routine use. Second, the retrospective nature inherently introduces potential biases. Postoperative pain scores were not collected at standardized intervals but during clinical visits. Measurement of imaging variables (hematometra and endometrioma size), while based on standard clinical protocols, may have interobserver variability. Unmeasured confounding, including potential variability in surgical technique detail and the possibility of postoperative anatomical re-obstruction, may influence recurrence. Pregnancy planning is a factor where fertility intentions could influence the follow-up patterns. Therefore, prospective validation through multicenter collaboration, incorporating diverse demographic cohorts, is warranted to verify the reproducibility and clinical applicability of the nomogram in the future. Third, while our overall cohort size is reasonable for model development, the number of patients reaching the very long-term follow-up milestones (e.g., 120 months) is smaller, resulting in wider confidence intervals for cumulative recurrence estimates at those time points. There is a risk of overfitting due to the low EPV, although the bootstrap validation (1,000 iterations) showed good calibration. Nevertheless, we used two complementary internal validation strategies (bootstrap and repeated K-fold cross-validation) to rigorously assess model performance within these constraints. The consistent results from both methods mitigate concerns regarding overfitting and support the model’s stability in our specific cohort.

In conclusion, we have developed and internally validated the nomogram for predicting long-term ovarian endometrioma recurrence in patients with OMA following corrective surgery. This model has the potential to guide personalized postoperative management by identifying high-risk individuals. Early anatomical correction and prolonged hormonal suppression emerge as optimal prevention strategies. However, its ultimate clinical utility awaits confirmation through external validation in independent, prospective cohorts.

## Data Availability

The raw data supporting the conclusions of this article will be made available by the authors, without undue reservation.
