# Supplementary material for: A predictive nomogram for postoperative ovarian endometrioma recurrence in patients with congenital obstructive Müllerian anomalies: a retrospective study
Source: Front Med (Lausanne). 2026 Feb 26;13:1714370. doi: 10.3389/fmed.2026.1714370 (PMC12979512; doi:10.3389/fmed.2026.1714370)
Supplement: Supplementary file 1 [file Data_Sheet_1.DOCX]

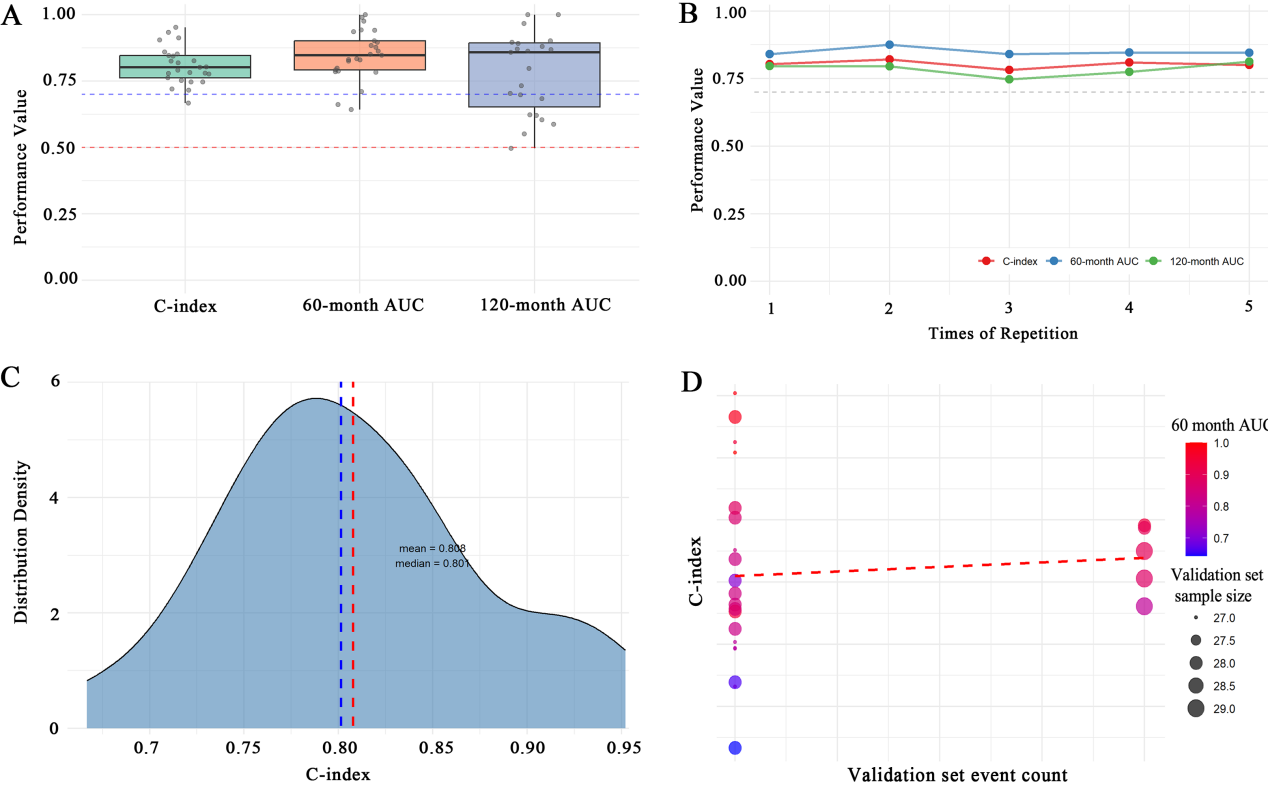


**Supplementary Figure S1.** Performance stability across repeated K-fold cross-validation. (A, B) Trajectories of C-index and time-dependent AUCs (60-month, 120-month) across 25 validation folds. (C, D) Performance metrics (C-index, 60-month AUC) plotted against validation set sample size and event count, demonstrating minimal fluctuation.
